# Different MRI-based radiomics models for differentiating misdiagnosed or ambiguous pleomorphic adenoma and Warthin tumor of the parotid gland: a multicenter study

**DOI:** 10.3389/fonc.2024.1392343

**Published:** 2024-06-13

**Authors:** Jing Yang, Qiu Bi, Yiren Jin, Yong Yang, Ji Du, Hongjiang Zhang, Kunhua Wu

**Affiliations:** ^1^ Department of MRI, The First People’s Hospital of Yunnan Province, The Affiliated Hospital of Kunming University of Science and Technology, Kunming, Yunnan, China; ^2^ Department of Radiation, The Cancer Hospital of Yunnan Province, The Third Affiliated Hospital of Kunming Medical University, Kunming, Yunnan, China

**Keywords:** parotid gland, MRI, radiomics, nomogram, pleomorphic adenoma, Warthin tumor

## Abstract

**Purpose:**

To evaluate the effectiveness of MRI-based radiomics models in distinguishing between Warthin tumors (WT) and misdiagnosed or ambiguous pleomorphic adenoma (PA).

**Methods:**

Data of patients with PA and WT from two centers were collected. MR images were used to extract radiomic features. The optimal radiomics model was found by running nine machine learning algorithms after feature reduction and selection. To create a clinical model, univariate logistic regression (LR) analysis and multivariate LR were used. The independent clinical predictors and radiomics were combined to create a nomogram. Two integrated models were constructed by the ensemble and stacking algorithms respectively based on the clinical model and the optimal radiomics model. The models’ performance was evaluated using the area under the curve (AUC).

**Results:**

There were 149 patients included in all. Gender, age, and smoking of patients were independent clinical predictors. With the greatest average AUC (0.896) and accuracy (0.839) in validation groups, the LR model was the optimal radiomics model. In the average validation group, the radiomics model based on LR did not have a higher AUC (0.795) than the clinical model (AUC = 0.909). The nomogram (AUC = 0.953) outperformed the radiomics model in terms of discrimination performance. The nomogram in the average validation group had a highest AUC than the stacking model (0.914) or ensemble model (0.798).

**Conclusion:**

Misdiagnosed or ambiguous PA and WT can be non-invasively distinguished using MRI-based radiomics models. The nomogram exhibited excellent and stable diagnostic performance. In daily work, it is necessary to combine with clinical parameters for distinguishing between PA and WT.

## 1 Introduction

Up to 80% of parotid tumors are benign with the two most common types being pleomorphic adenoma (PA) and Warthin tumor (WT) (1, 2). Compared with WT, PA exhibits a higher potential for malignant transformation and recurrence, so the surgical approaches and prognosis are completely different (3, 4). Hence, for the purpose of precisely and individually treating patients with benign parotid tumors, it is crucial to accurately distinguish between PA and WT.

At present, the preoperative diagnosis of PA or WT relies on fine needle aspiration cytology (FNAC) and radiological images. However, FNAC is not always conclusive because of sampling difficulties and the experience of pathologist (5, 6). Furthermore, FNAC is invasive, which may lead to hemorrhage (7), inflammation (8), and dissemination of tumor cells along the needle route (9). Patients with similar clinical factors may have varying outcomes, and it is often difficult to definitively distinguish between WT and PA based solely on clinical factors. In comparison to CT and needle biopsy, MRI offers several advantages such as non-invasiveness, absence of radiation, and excellent soft tissue resolution (10). In the evaluation of parotid tumors, MRI can provide information about the size, location, shape, and characteristics of the tumor, which can help guide treatment decisions (11, 12). Nonetheless, conventional MRI differential diagnosis has not always been adequate because of the substantial overlap in morphological features between PA and WT. In addition, conventional MRI diagnosis may have a subjective component and depend on the expertise and experience of radiologist (13).

Radiomics can extract high throughput of quantitative features by converting images into amenable data, and the analyzing these data for decision support (14). Radiomics can provide much more comprehensive information from medical images than human eyes (15). In recent years, radiomics has been widely used for preoperative diagnosis of parotid tumors (16–18). Some previous studies have tried to discriminate benign and malignant parotid tumors using radiomics (19, 20), but only a few of them have analyzed the differentiation of PA from WT (21, 22). However, there is no research focused on differentiating misdiagnosed or ambiguous PA and WT using radiomics.

Therefore, we use a variety of machine learning methods to establish different MRI-based radiomics models and determine the optimal radiomics models for identifying misdiagnosed or ambiguous PA and WT. By integrating a variety of models combining radiomics and clinical parameters, we evaluate the effect of multimode combined application in differential diagnosis of the disease, so as to improve the accuracy of diagnosis of the disease.

## 2 Materials and methods

### 2.1 Study population

**Figure 1:**
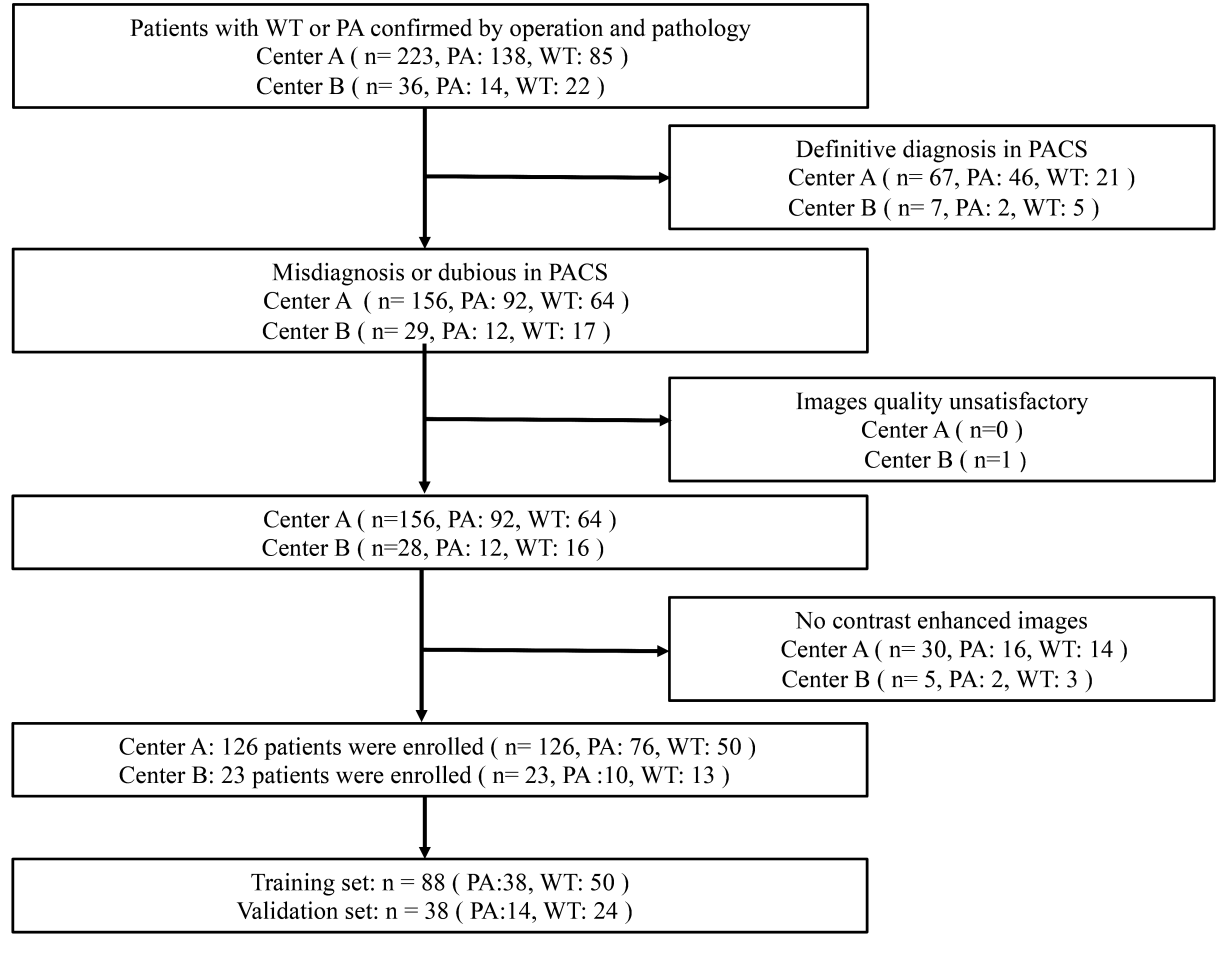
Flowchart for selecting the study population. PA, pleomorphic adenomas; WT, Warthin tumors.

The ethical approval of two clinical centers approved this retrospective study. The informed consent was waived. All the enrolled patients with PA or WT were from centers A and B between January 2015 and June 2022. The inclusion criteria were as follows: (1) patients with WT or PA confirmed by operation and pathology; (2) PA and WT were diagnosed as misdiagnosed or ambiguous on Picture Archiving and Communication Systems (PACS); (3) complete clinical data; patients with satisfactory image quality; (4) underwent MR examination no more than 7 days before surgery. The exclusion criteria were as follows: (1) underwent parotid puncture, surgery, or chemoradiotherapy before MR examination; (2) PA and WT were identified on the PACS; (3) image quality unsatisfactory due to motion artifacts or false teeth artifacts, etc.; (4) absence of enhanced images. A total of 126 patients (76 PA, 50 WT) were assigned to training group (88 patients) and internal verification group (38 patients) and center B (23 patients) as external verification group according to 4:1. A follow diagram of the study population is shown in **Figure 1**. The clinical characteristics included gender, age, smoking history, lesion mobility, and lesion hardness were collected.

### 2.2 MRI image acquisition

All MR examinations were performed using 1.5/3.0-T scanners (Philips 1.5 T, Siemens Aera 1.5T, and Siemens Prisma 3.0T, GE Signa HDxt (3.0T). All patients underwent a preoperative MR examination using parotid scan protocol. Parameter details are shown in **Table 1**. The contrast-enhanced images were obtained after administered (0.1 mmol/kg) at a rate of 2.0 mL/s via the elbow vein.

**Table 1:** The parameter details of primary sequences.

	Sequence	Repetition time (ms)	Echo time (ms)	Field of view (mm^2^)	Acquisition matrix (ms)	Slice thickness (mm)	Slice gap (mm)
Philips 1.5 T	T2WI	2,000	90	240×240	268×200	4	1
	T1WI	Shortest	Shortest	240×200	268×200	4	−2
	CE-T1WI	Shortest	Shortest	240×220	268×220	4	−2
Siemens Aera 1.5 T	T2WI	5,600	80	240×240	224×320	5	1
	T1WI	7.11	2.39	240×240	240×320	3	0.6
	CE-T1WI	7.11	2.39	240×240	240×320	3	0.6
Siemens Prisma 3.0 T	T2WI	4,800	83	220×220	240×320	4	1
	T1WI	6.18	2.46	220×220	259×288	3	1
	CE-T1WI	6.18	2.46	220×220	256×320	3	0.6
GE Signa HDxt 3.0T	T2WI	4,740	68	260×260	288×224	5	1
	T1WI	5.7	1.4	260×260	240×224	4	1
	CE-T1WI	5.7	1.4	260×260	264×224	4	1

T2WI, T2-weighted imaging; T1WI, T1-weighted imaging; CE-T1WI, contrast-enhanced T1-weighted imaging.

### 2.3 Conventional MRI features

The MRI features were assessed by two radiologists (reader 1 with 8 years of experience in neck MRI and reader 2 with 6 years of experience in neck MRI). The radiologist was blinded to the clinical data and the histological results. The MRI features were as follows: (1) tumor location (left side or right side, superficial lobe or deep lobe of parotid); (2) tumor diameter (craniocaudal, transverse, and anteroposterior diameter); (3) lobulated appearance, cystic degeneration and capsule (absent or present) (13), (4) tumor margin (clear or unclear) (23); (5) “hamming sign,” which means tumor margin thin band or petal high signal on T2-weighted imaging (T2WI), more than 1/4 of the circumference of the same layer (24); (6) tumor homogeneity on T1-weighted imaging (T1WI), T2WI, and contrast-enhanced T1-weighted imaging (CE-T1WI) (24, 25).

### 2.4 Image segmentation

MRI images of axial T2WI, T1WI, and CE-T1WI were stored in Digital Imaging and Communications in Medicine (DICOM) format and uploaded into 3D Slicer 4.11.0 software (https://www.slicer.org/). The segmentation of the tumors was performed by two radiologists (reader 1 and reader 2), who were blinded to the clinical information and histopathological results. The region of interest (ROI) of the lesion was manually delineated layer by layer to cover the whole tumor as largely as possible (including cystic and necrotic areas) but avoiding normal tissue to form a three-dimensional (3D) volume of interest (VOI). Reader 1 draws the ROI. Two months later, two readers (reader 1 and reader 2) had a brief review in the same case.

### 2.5 Image preprocessing and feature extraction

Pyradiomics (https://pypi.org/project/pyradiomics/) is an open-source Python software that was used for image preprocessing and feature extraction. The voxel size of 1 × 1 × 1 mm^3^ was resampled in order to improve the comparability of the MRI gray-level values (26). To standardize image intensity, the gray-level values in the photographs were spread across the range of 0–600. There were 5,343 radiomics features obtained for every patient out of the total 1,781 features that were extracted from each MRI sequence. Z score was used to standardize all of the aforementioned features.

### 2.6 Feature selection

The training group’s patient datasets for WT and PA were balanced by the application of the synthetic minority oversampling technique. For every feature, the intraclass correlation coefficient (ICC) was computed. Selection was made of features with ICC values ≥0.75 for both observers within and between. In order to determine whether features were redundant, Pearson correlation coefficients were obtained. When two features had a correlation coefficient of less than 0.9, the feature with the highest mean absolute correlation was eliminated. To find the most representative features, we employed a least absolute shrinkage and selection operator (LASSO) regression model and 10-fold cross-validation (27).

### 2.7 Models’ construction

#### 2.7.1 Clinical model

The differences in clinical parameters and conventional MRI features between PA and WT in the training group were compared using univariate analysis, and the clinical factors and MRI features with significant difference were determined. The univariate logistic regression (LR) analysis and multivariate LR were used to construct clinical model and find out clinical predictors.

#### 2.7.2 Radiomics model

In this study, nine mainstream machine learning algorithms were used to build radiomics models for distinguishing PA and WT, which included logistic regression (LR), K-nearest neighbor (KNN), support vector machine (SVM), random forest (RF), stochastic gradient descent (SGD), extremely randomized trees (ET), decision tree (DT), eXtreme Gradient Boosting (XGBoost), and Light Gradient Boosting Machine (LightGBM). In both the internal and external validation groups, the nine machine models’ diagnostic performances were assessed based on sensitivity, specificity, accuracy, and the area under the curve (AUC) of the receiver operating characteristic curve (ROC). The radiomics model with the highest average AUC was chosen as the optimal model. A radiomics score (radscore) was calculated for each patient.

#### 2.7.3 Fusion model

A nomogram integrating independent clinical parameters and the radscore was constructed using multivariate LR analysis.

Using a meta-regression model to integrate many models, the stacking model, which is an ensemble learning technology that increases the accuracy of result prediction, was employed. A two-tier stacking model was used to calculate. The first tier used the predicted results of the clinical model and the optimal radiomics model, and the second tier used the results of the first tier as the input of the multivariate LR. These input properties were integrated using the meta-regressor to achieve model fusion (28).

Utilizing super learner, an integrated technique, the ensemble algorithm is developed (29). By employing the weighted average approach to derive the anticipated values from both the clinical model and the optimal radiomics model, the new output was ultimately utilized as the outcome.

**Figure 2:**
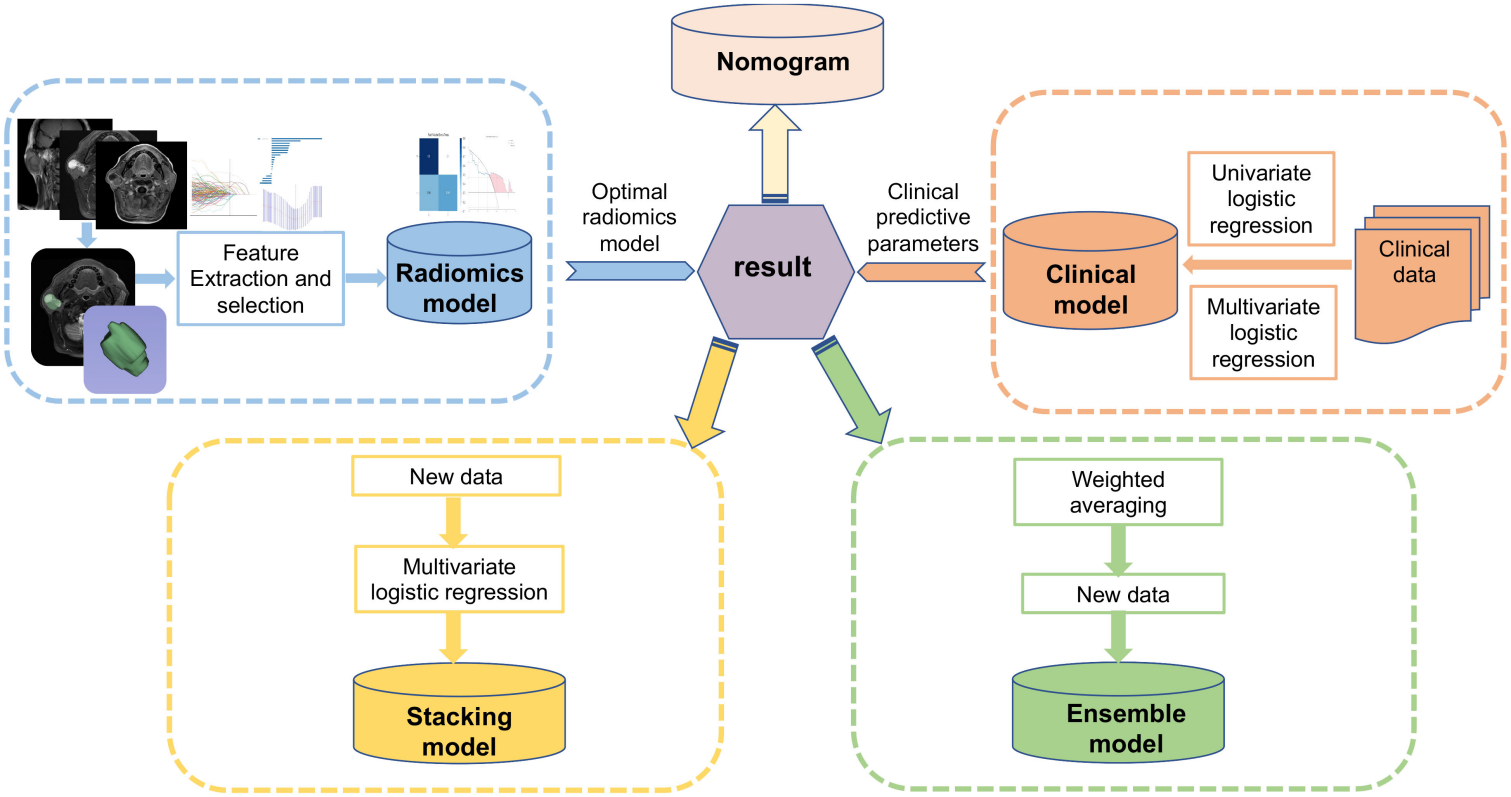
Workflow of this study.

The Python (https://www.python.org/getit/) was used to perform the above model building, and **Figure 2** illustrates the detailed process of model structure. To evaluate the effectiveness and goodness of fit of each model, metrics such as sensitivity, specificity, accuracy, and the area under the curve (AUC) of the calibration curve and receiver operating characteristic curve (ROC) were employed.

### 2.8 Clinical application of the models

To diagnose PA and WT in the training and validation groups, one radiologist solely reviewed the MR images while being blind to the histological results and clinical information. The radiologist’s AUC, accuracy, specificity, and sensitivity were calculated. The clinical usefulness and net benefit of the radiologist and various models were estimated using the net reclassification index (NRI), integrated discrimination index (IDI), and clinical decisive curve (CDC).

### 2.9 Statistical analyses

Statistical analysis was conducted with SPSS 26.0 (IBM, New York, USA), R software 4.1.2 (https://www.r-project.org/), and Python 3.9.7 (https://www.python.org/). The mean value ± standard deviation and counts were used to express categorical variables and continuous data, respectively.

The continuous data distribution was examined for normalcy using the Kolmogorov–Smirnov test. One-way ANOVA or the Kruskal–Wallis test was used to evaluate continuous variables, whereas the Chi-square test or Fisher’s exact test was used to investigate categorical variables. Both univariate and multivariate LR analyses were employed in the model building and clinical predictor filtering processes. At *p* < 0.05, statistical significance was established. Pearson correlation analysis was used to evaluate the correlations between continuous variables, whereas Spearman correlation analyses were used to investigate the relationships between continuous variables and ranking data. It is considered to be correlations between the variables if *p* < 0.05. DeLong test was used to compare the prediction performance of different models. At *p* < 0.05, statistical significance was established.

## 3 Results

### 3.1 Clinical parameters

The MRI characteristics and basic demographic information of the patients are given in **Table 2**. Age, gender, and smoking may be utilized to predict WT and PA, according to univariate logistic regression analysis. Multivariate LR analysis showed that gender, age, and smoking remained as independent predictors in the clinical factor model.

**Table 2:** Clinical and conventional imaging characteristics for patients.

		Training group	Internal validation group	External validation group	*p-value*
Total number		88	38	23	
Age		46.09 ± 13.91	50.13 ± 15.52	52.43 ± 14.14	0.11
Gender	Female	31 (35.2%)	10 (26.3%)	4 (17.4%)	0.21
	Male	57 (64.8%)	28 (73.7%)	19 (82.6%)	
Smoking	Yes	43 (48.9%)	19 (50.0%)	15 (65.2%)	0.37
	No	45 (52.1%)	19 (50.0%)	8 (34.8%)	
Pian	Yes	1 (1.14%)	1 (2.63%)	2 (8.7%)	0.14
	No	87 (98.9%)	37 (97.4%)	21 (91.3%)	
Lesion mobility	Yes	61 (69.3%)	23 (60.5%)	20 (87.0%)	0.09
	No	27 (30.7%)	15 (39.5%)	3 (13.0%)	
Lesion consistency	Soft	7 (9.95%)	4 (10.5%)	5 (21.7%)	0.19
	Hard	81 (92.0%)	34 (89.5%)	18 (78.3%)	
Side	Right	50 (56.8%)	22 (57.9%)	12 (52.2%)	0.90
	Left	38 (43.2%)	16 (42.1%)	11 (47.8%)	
Location	Superficial lobe	87 (98.9%)	38 (100%)	23 (100%)	1.00
	Deep lobe	1 (1.14%)	0 (0.00%)	0 (0.00%)	
Lobulated border	Yes	39 (44.3%)	13 (34.2%)	10 (43.5%)	0.56
	No	49 (55.7%)	25 (65.8%)	13 (56.5%)	
Cystic degeneration	Yes	42 (47.7%)	21 (55.3%)	6 (26.1%)	0.08
	No	46 (52.3%)	17 (44.7%)	17 (73.9%)	
Margin	Clear	83 (94.3%)	37 (97.4%)	23 (100%)	0.60
	Unclear	5 (5.68%)	1 (2.63%)	0 (0.00%)	
Capsule	Present	83 (94.3%)	37 (97.4%)	23 (100%)	0.60
	Absent	5 (5.68%)	1 (2.63%)	0 (0.00%)	
“Hemming Sign”	Present	26 (29.5%)	11 (28.9%)	2 (8.70%)	0.12
	Absent	62 (70.5%)	27 (71.1%)	21 (91.3%)	
T1WI homogeneity	Yes	65 (73.9%)	28 (73.7%)	14 (60.9%)	0.45
	No	23 (26.1%)	10 (26.3%)	9 (39.1%)	
T2WI homogeneity	Yes	28 (31.8%)	18 (47.4%)	10 (43.5%)	0.21
	No	60 (68.2%)	20 (52.6%)	13 (56.5%)	
CE-T1WI homogeneity	Yes	15 (17.0%)	6 (15.8%)	10 (43.5%)	0.05
	No	73 (83.0%)	32 (84.2%)	13 (56.5%)	
Craniocaudal diameter		2.72 ± 1.15	2.92 ± 1.28	3.13 ± 1.09	0.29
Transverse diameter		2.23 ± 0.94	2.32 ± 0.90	2.56 ± 0.83	0.31
Anteroposterior diameter		2.21 ± 0.85	2.18 ± 0.97	2.14 ± 0.62	0.91

### 3.2 Feature selection and performance of different machine learning models

**Figure 3:**
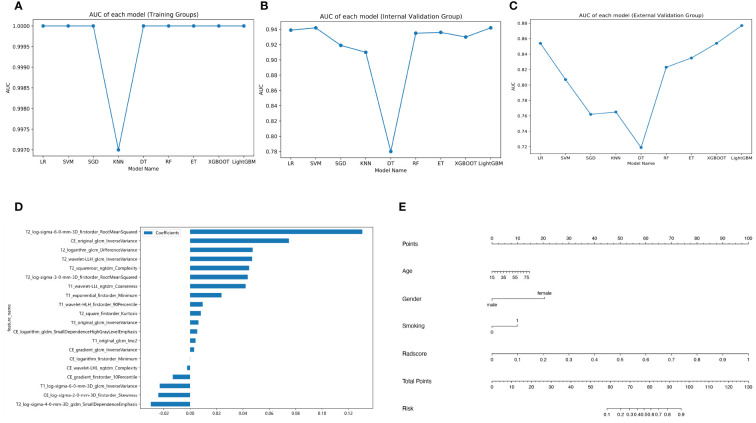
Different model building. Broken line graphs of the area under the curve (AUC) for different machine learning algorithms in the training group **(A)**, the internal validation group **(B)**, and the external validation group **(C)**. Bar chart of feature weight for the logistic regression model **(D)**. Nomogram of the training group **(E)**.

Out of all the extracted features, 3,836 features were excluded due to the ICC values less than 0.75 either between or within observers. Following the completion of the Pearson correlation analysis, 605 features were retained. There were then 20 features identified by the LASSO classifier (**Supplementary Material 1**). **Table 3** displays the AUC, accuracy, sensitivity, and specificity of radiomics models building by nine machine learning algorithms. **Figures 3A–C** show broken line graphs of AUC for various algorithms in the training, internal validation, and external validation groups. With an AUC of 0.896, and an accuracy of 0.839 in the average validation groups, the LR algorithm was the best radiomic model. Consequently, it was thought that the LR algorithm was the best option for building radiomics models.

**Table 3:** The performance of various machine learning algorithms.

	Training group		Internal validation group		External validation group		Average validation groups	
	AUC	Accuracy	AUC	Accuracy	AUC	Accuracy	AUC	Accuracy
LR	1.000	1.000	0.939	0.895	0.854	0.783	0.896	0.839
SVM	1.000	1.000	0.942	0.895	0.807	0.782	0.875	0.839
SGD	1.000	1.000	0.919	0.842	0.762	0.696	0.840	0.769
KNN	0.997	0.955	0.910	0.895	0.765	0.696	0.838	0.795
DT	1.000	1.000	0.780	0.790	0.719	0.696	0.749	0.743
RF	1.000	1.000	0.935	0.895	0.823	0.696	0.879	0.795
ET	1.000	1.000	0.936	0.921	0.835	0.696	0.885	0.809
XGBoost	1.000	1.000	0.930	0.895	0.854	0.652	0.892	0.773
LightGBM	1.000	1.000	0.942	0.921	0.877	0.609	0.909	0.765

AUC, area under the curve; LR, logistic regression; SVM, support vector machine; SGD, stochastic gradient descent; KNN, K nearest neighbor; DT, decision tree; RF, random forest; ET, extremely randomized trees; XGBoost, eXtreme Gradient Boosting; LightGBM, Light Gradient Boosting Machine.

The coefficients and intercepts derived from the LR model were used to calculate the radscore. **Figure 3D** displays the selected features and weights.

### 3.3 Different fusion models: performance and clinical applications

**Figure 4:**
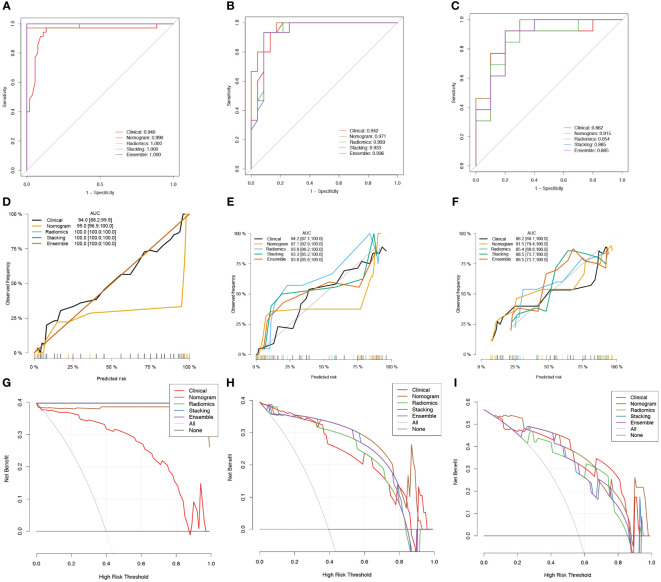
Receiver operator characteristic (ROC) curves **(A-C)**, calibration curves **(D-F)**, and clinical decision curves (CDCs) of different models in the training group **(A, D, G)**, the internal validation group **(B, E, H)**, and the external validation group **(C, F, I)**.

The radscore and the clinical predictive characteristics (smoking, age, and gender) were used to construct a nomogram (**Figure 3E**). The diagnostic performance of each model is presented in **Table 4**. ROC curves and calibration curves of different models are shown in **Figures 4A–F**. In the training group, the clinical, radiomics, nomogram, stacking, and ensemble models’ AUCs were 0.940, 1.00, 0.990, 1.00, and 1.00. They were, in order, 0.942, 0.939, 0.971, 0.936, and 0.936 in the internal validation group. They were, in order, 0.862, 0.854, 0.915, 0.885, and 0.885 in the external validation group. They were, in the average validation group, 0.909, 0.795, 0.953, 0.914, and 0.798, in that order. The AUC of the nomogram was the highest in the average validation group. To perform the Delong test in the average validation group, we merged the data from the external and internal validation group. The DeLong test showed that the prediction performance of the nomogram was significantly better. There was a statistical difference between nomogram and ensemble model, and between nomogram and radiomics model (*P* < 0.05). There was no statistical difference between nomogram and stacking model (*P* = 0.075), as well as nomogram and clinical model (*P* = 0.163) (**Supplementary Material 2**).

**Table 4:** Diagnostic efficiency and clinical benefit of different models.

	Models	AUC	Accuracy	Sensitivity	Specificity	NRI	IDI
Training group	Clinical model Radiomics model Nomogram Stacking model Ensemble model	0.940 1.000 0.990 1.000 1.000	0.909 1.000 0.989 0.989 0.989	0.914 1.000 0.971 0.971 0.971	0.906 1.000 1.000 1.000 1.000	0.545 0.545 0.523 0.522 0.523	0.528 0.783 0.714 0.743 0.655
Internal validation group	Clinical model Radiomics model Nomogram Stacking model Ensemble model	0.942 0.939 0.971 0.936 0.936	0.942 0.894 0.895 0.921 0.921	1.000 0.867 0.933 0.933 0.933	0.739 0.913 0.870 0.913 0.913	0.474 0.316 0.368 0.368 0.368	0.237 0.413 0.383 0.379 0.307
External validation group	Clinical model Radiomics model Nomogram Stacking model Ensemble model	0.862 0.854 0.915 0.885 0.885	0.826 0.783 0.826 0.696 0.739	0.846 0.692 0.846 0.615 0.615	0.800 0.900 0.800 0.800 0.900	0.869 0.782 0.869 0.695 0.695	1.301 1.294 1.334 1.272 1.227
Average validation group	Clinical model Radiomics model Nomogram Stacking model Ensemble model	0.909 0.795 0.953 0.914 0.798	0.623 0.311 0.902 0.885 0.361	0.536 0.286 0.964 0.929 0.429	0.697 0.333 0.848 0.848 0.303	0.933 0.319 1.513 0.692 0.977	0.215 0.001 0.668 0.564 -0.053

AUC, area under the curve; NRI, net reclassification index; IDI, integrated discrimination index.

**Figures 4G–I** displays the CDCs for each model, whereas **Table 4** presents the NRI and IDI. The nomogram model had a highest NRI (1.513) and IDI (0.668) than other models in the average validation group. It shows that nomogram had good ability for the differentiation of PA and WT than the other models.

## 4 Discussion

We found that gender, age, and smoking were clinical independent predictors for the differential diagnosis of PA and WT. The LR algorithm model, which was based on nine popular machine learning algorithms, was the best radiomics model with the highest accuracy and AUC. The fusion models—nomogram, stacking, and ensemble—also demonstrated superior diagnostic performance and produced a good net clinical benefit when compared with the clinical model. In comparison with the best radiomics model, the nomogram showed a better AUC. It also outperformed stacking and ensemble models in terms of superior generalization ability and more consistent discrimination efficiency.

Previous studies have reported that gender, age, and smoking history of patients had significance in the identification of PA and WT (30, 31). Our results were similar to those studies. Some studies suggested that duration of smoking was a strong risk factor (32). Because male smokers were more prevalent, WT was more common in men. The pathogenesis may relate to the fact that tobacco contains chemical irritants such as benzopyrene, arsenic, and N-nitrosoguanidine (31).. These irritants leading to secondary tumor change was a lengthy phenomenon, so WT occurred in middle or old age. Some studies suggested that the comparatively significantly greater incidence of WT in men might indicate a hormone dependence, and progesterone receptors have been found in WT (33). The evidence of progesterone receptor in WT implicated a potential role of endocrine factors in the development of this tumor, which might explain the predominance of the male sex regarding this disease (34).

**Figure 5:**
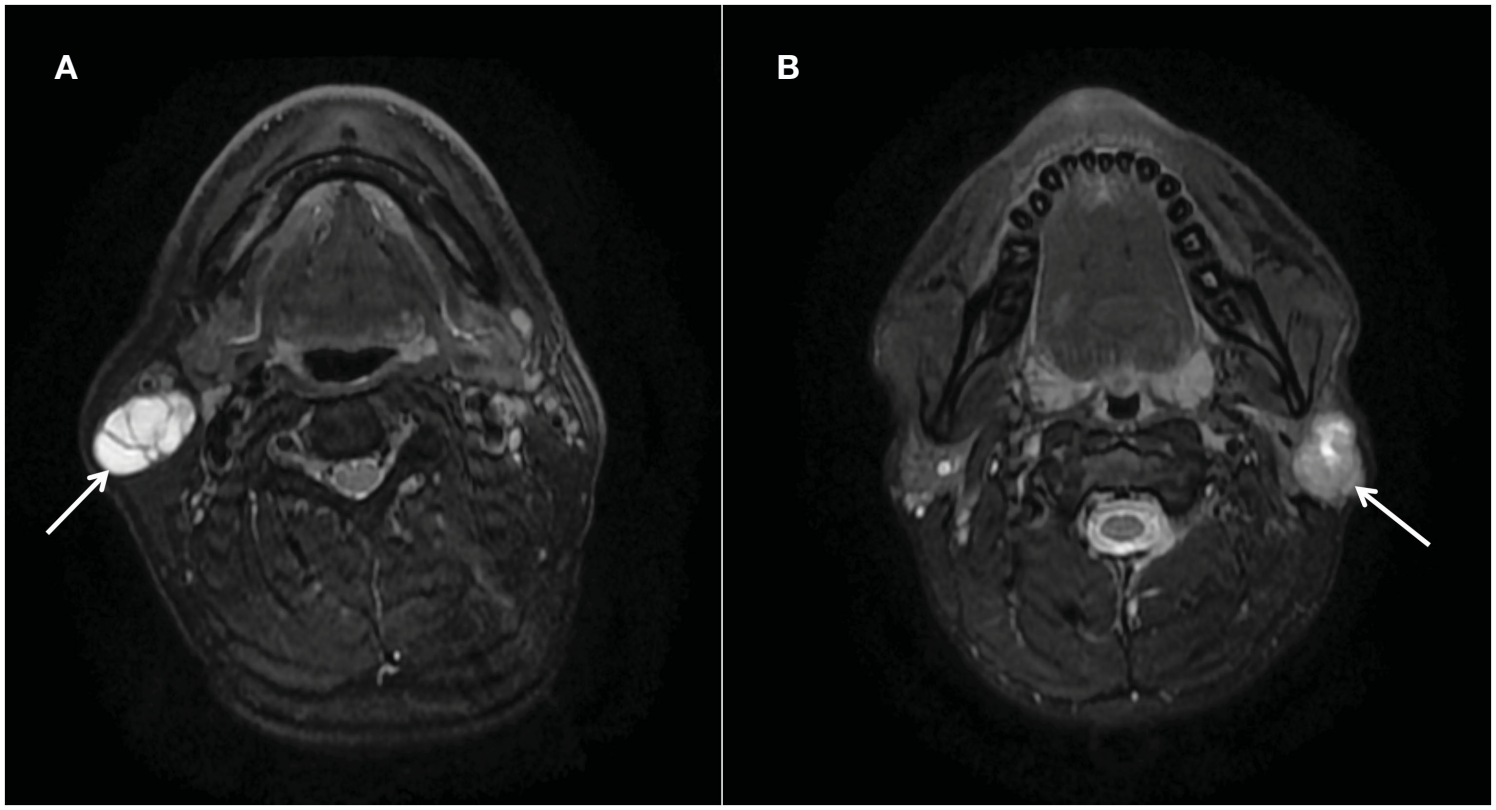
**(A)** Warthin tumors (arrows) in the right parotid gland of a 52-year-old man. T2-weighted image (axial plane) shows a markedly high-intensity tumor; a partition is visible within it. **(B)** Pleomorphic adenoma (arrows) in the left parotid gland of a 26-year-old man. T2-weighted image (axial plane) shows a slightly hypointense tumor. There are irregular areas of high intensity in the upper part of the tumor.

PAs are also known as mixed tumors due to histological heterogeneity, which also suggests that it is represented by various imaging findings (23). When PA has fewer cellular components of mucoid tissue, high signal intensity on T2WI images decreases, which reduces the rate depending on the proportion of cellular components (35).The tumor signal expression of WT depends mainly on the cystic component of the tumor, and as the size of the cystic component increases, the internal structure looks bright at T2WI images that may simulate PA (36). When PA and WT showed similar imaging manifestations, it was difficult to distinguish PA from WT based on conventional MR imaging (**Figure 5**). The radiologists only paid attention to the imaging manifestations of the tumor while ignoring the clinical characteristics, which was more likely to be misdiagnosed.

Radiomics is a non-invasive technique that builds models from digitized medical images and uses clever computation to convert them into high-dimensional, quantitative data that can be used to improve medical decision-making and provide useful diagnoses (14). Liu et al. (37) reported that there were no appreciable variations between MRI and CT in radiomics characteristics for diagnosing parotid malignancies. In this study, the diagnostic efficacy of the radiomics model was not as good as that of the clinical model. Potential explanations for these results included the subjective impact of individual clinical experience as well as a single imaging index. T2WI provided the vital features for the optimal radiomics model. PA contains mucoid tissue and usually shows a high signal on T2WI (38). In comparison, WT are epithelial tissues with lymphoid hyperplasia that contain cystic components of approximately 30% protein liquids or viscous colloids, and they usually show a hypointense/with hyperintensity signal on T2WI (36). Additionally, this study found that GLCM features could help discriminate between PA and WT, similar to the results of Gabelloni (39). The coarseness of the texture was represented by the zone percentage of GLCM features, which may more accurately capture the heterogeneity of various tumor types.

Recognizing the best machine learning techniques for radiomics models is essential (40). Thus, we employed nine common classification algorithms in model construction. LR outperformed other classifiers, which were consistent with the results of Lu et al. (41). More training samples may have been needed for sophisticated models, which could be the cause (42). The optimal radiomics model based on LR did not have a higher AUC than the clinical model. This result also illustrates that when there is a problem with the observation of traditional imaging, radiologists should combine clinical data. Evidence-based clinical decision support systems can be produced with accuracy and dependability by combining radiomics features with clinical parameters and other pertinent data (43). In this study, the clinical or radiomics model did not perform as well in terms of diagnostic performance and clinical net benefits as the nomogram, stacking model, and ensemble model, which are instances of fusion models constructed utilizing clinical parameters and radiomics features. Additionally, the nomogram exhibited a highest AUC when compared with the other models. Zheng et al. (44) constructed a radiomics nomogram based on MRI that had good prediction efficiency in distinguishing PA from WT, obtaining a similar conclusion as this research. The ensemble strategy has the advantage of being able to reduce the variance and bias of the model while also enhancing its robustness and generalization in classification and prediction, by using a strong majority voting or group average method (45). A recent report had proposed that the stacking ensemble model obtained excellent diagnostic performance and showed good stability of the calibration plot (46). While AUCs for the ensemble and stacking models were less than those of the nomogram in the current study, their diagnostic performance in the average validation groups was comparable with and superior to that of the radiomics models. As a result, the nomogram demonstrated better and more consistent differential diagnosis efficiency with superior reproducibility and reliability when compared with stacking and ensemble models.

This study’s limitations were the fact that it only included participants from two centers, and the sample of external test data was relatively small. Additional patients from more centers must be included to expand the universality in clinical applications, in the future. Second, this was a retrospective study, which might cause potential selection bias. In the future, prospective validation will be performed. Third, there were variations in the MRI scanner and parameters, which could have an impact on the models’ output. We performed the N4 bias field correction. Fourth, we only studied conventional MRI sequences, with limited interpretability. Other quantitative functional MRI sequences, such as DWI and DCE-MRI, still need to be further explored.

## 5 Conclusions

The MRI-based radiomics models can be accomplished to preoperatively differentiate misdiagnosed or ambiguous PA and WT, and the LR algorithm-established model is the optimal radiomics model. The nomogram is an effective tool for preoperative and non-invasive distinguishing PA and WT, which can be challenging for radiologists and surgeons to ascertain prior to surgery. In daily work, it is necessary to combine with clinical parameters such as gender, age, and smoking when radiologists are difficult to distinguish PA from WT.

## Data availability statement

The raw data supporting the conclusions of this article will be made available by the authors, without undue reservation.

## Ethics statement

The studies involving humans were approved by the First People’s Hospital of Yunnan Province. The studies were conducted in accordance with the local legislation and institutional requirements. The ethics committee/institutional review board waived the requirement of written informed consent for participation from the participants or the participants’ legal guardians/next of kin because this is a retrospective study, so informed consent was waived by the ethics committee.

## Author contributions

JY: Methodology, Writing – original draft. QB: Conceptualization, Methodology, Writing – review & editing. YJ: Validation, Writing – review & editing. YY: Data curation, Writing – review & editing. JD: Data curation, Writing – review & editing. HZ: Writing – review & editing. KW: Writing – review & editing.

## Supplementary material

Supplemental material 1 Feature selection using the least absolute shrinkage and selection operator (LASSO) regression model. The cross-validation plot **(A)** and the coefficient profile plot **(B)**.

Supplemental material 2 Feature training group, internal validation group, external validation group and average validation group of Delong test **(A–D)**.
