# Supplementary figures and images for: Different MRI-based radiomics models for differentiating misdiagnosed or ambiguous pleomorphic adenoma and Warthin tumor of the parotid gland: a multicenter study
Source: Front Oncol. 2024 Jun 13;14:1392343. doi: 10.3389/fonc.2024.1392343 (PMC11208325; doi:10.3389/fonc.2024.1392343)

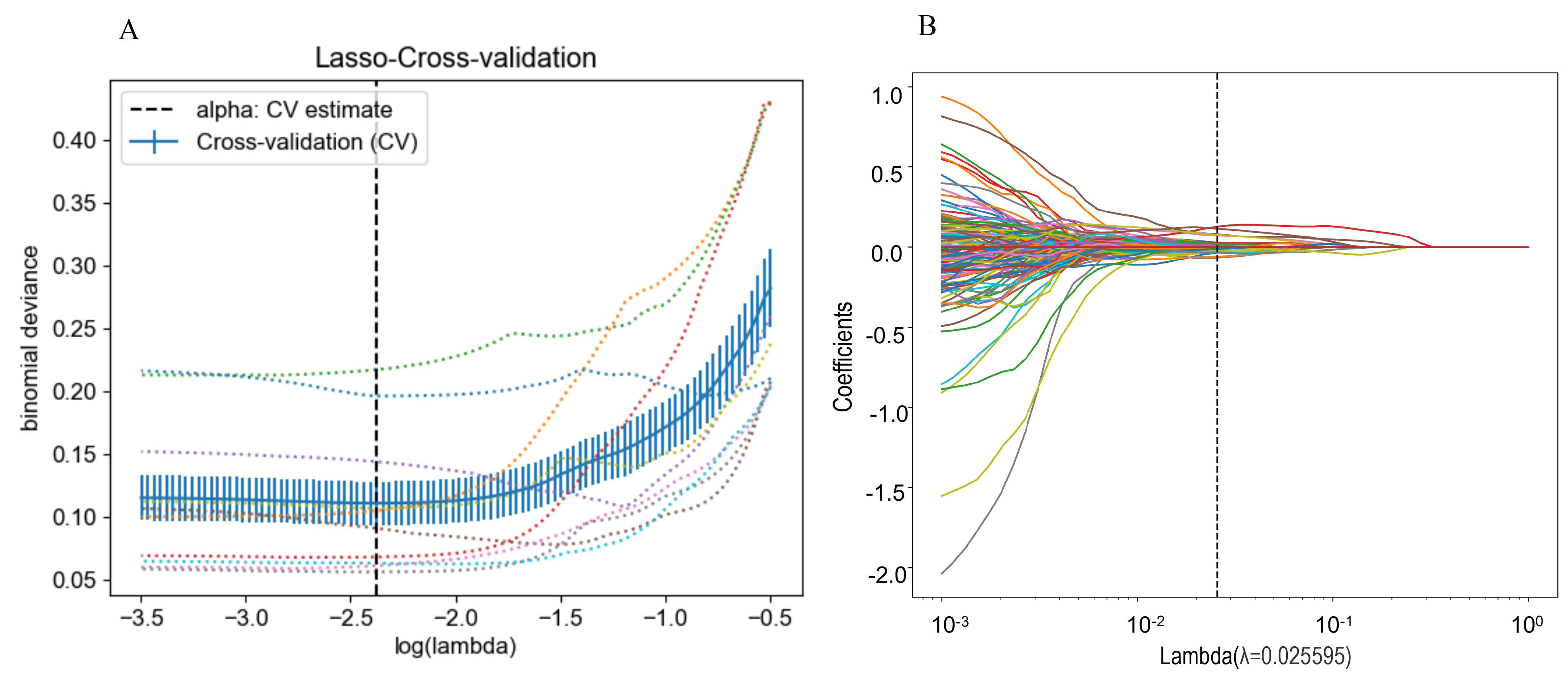

Supplement: Supplemental material 1 — Feature selection using the least absolute shrinkage and selection operator (LASSO) regression model. The cross-validation plot (A) and the coefficient profile plot (B). [file Image_1.tif]

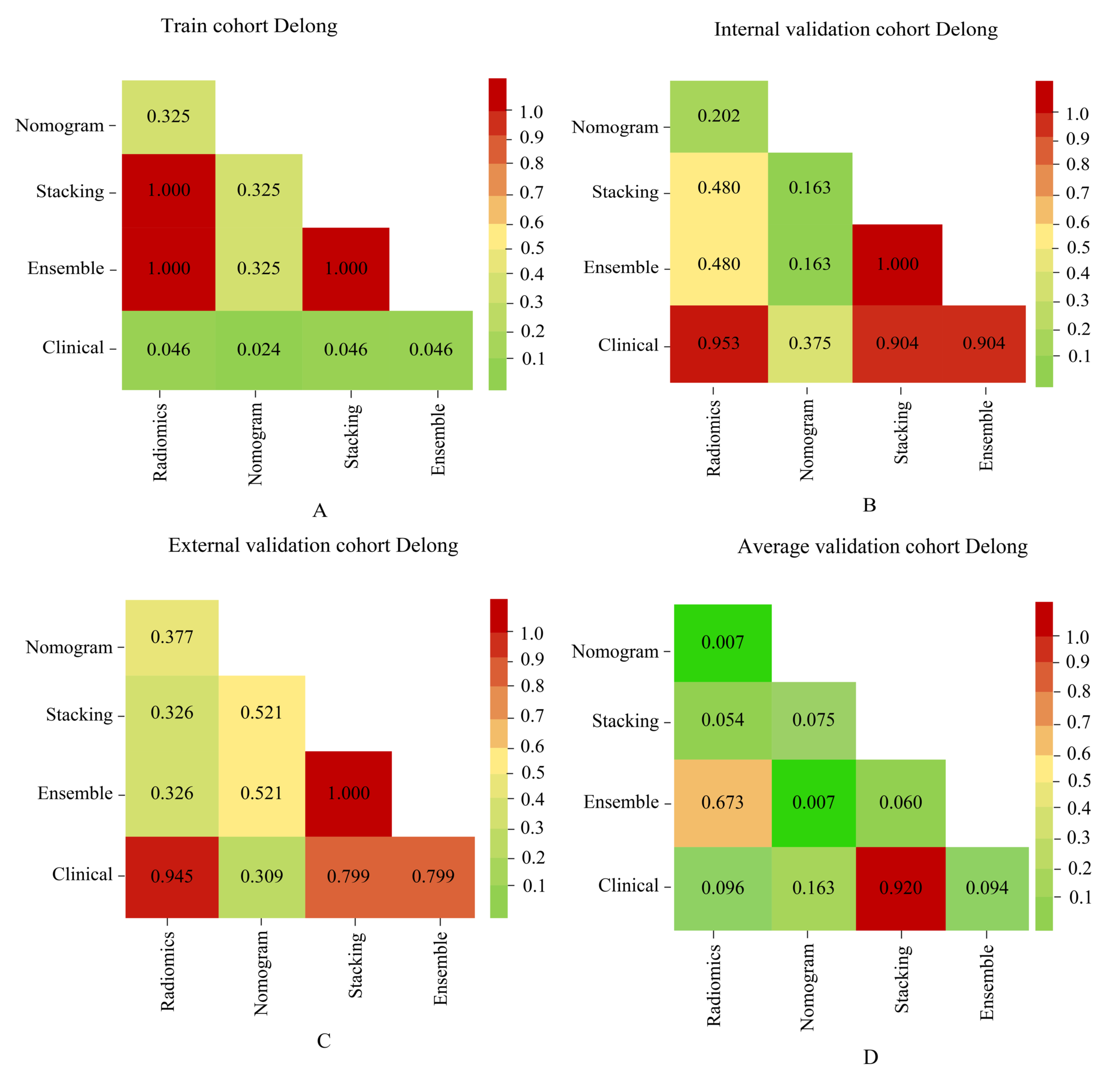

Supplement: Supplemental material 2 — Feature training group, internal validation group, external validation group and average validation group of Delong test (A–D). [file Image_2.tif]
